# Prostate cancer mortality in Brazil 1990-2019: geographical distribution and trends

**DOI:** 10.1590/0037-8682-0277-2021

**Published:** 2022-01-28

**Authors:** Daniel Albrecht Iser, Guilherme Ranzi Cobalchini, Max Moura de Oliveira, Renato Teixeira, Deborah Carvalho Malta, Mohsen Naghavi, Betine Pinto Moehlecke Iser

**Affiliations:** 1 Universidade do Sul de Santa Catarina, Programa de Pós-Graduação em Ciências da Saúde, Tubarão, SC, Brasil.; 2 Universidade do Sul de Santa Catarina, Faculdade de Medicina, Tubarão, SC, Brasil.; 3 Universidade Federal de Goiás, Instituto de Patologia Tropical e Saúde Pública, Departamento de Saúde Coletiva, Goiânia, GO, Brasil.; 4 Universidade Federal de Minas Gerais, Belo Horizonte, MG, Brasil.; 5 University of Washington, Institute for Health Metrics and Evaluation, Seattle, WA, United States.

**Keywords:** Prostate neoplasms, Mortality, Trends, Epidemiology

## Abstract

**INTRODUCTION:**

To analyze the trend of prostate cancer mortality in the Brazilian population of 40 years of age and above.

**METHODS:**

Time series ecological study of the mortality rates due to prostate cancer in men of 40 years of age and above, using data from the Global Burden of Disease 2019 (GBD). Age-standardized mortality rates were calculated, as well as the age-standardized rates by the GBD for the global population, per 100,000 inhabitants, for Brazil and its States, from 1990 to 2019. The annual average percent change (AAPC) was calculated to identify the mortality trends in Brazil, through linear regression using the Joinpoint Regression Program.

**RESULTS:**

The standardized rates of prostate cancer mortality in Brazil were 76.89 in 1990 and 74.96 deaths for every 100 thousand men ≥ 40 years of age in 2019, with a stability trend. By age group, it was observed a decreasing trend up to 79 years of age, and an increasing trend as of 80 years of age. The state of Bahia showed the highest increase in mortality in the period (1.2%/year), followed by Maranhão and Pernambuco (1.0 and 0.9%/year). A decrease of prostate cancer mortality was found in the Federal District, Goiás, Minas Gerais, Rio de Janeiro, Rio Grande do Sul, Roraima, Santa Catarina, São Paulo, and Sergipe.

**CONCLUSIONS:**

In Brazil, the standardized mortality rates show a trend toward stability from 1990 to 2019 and no pattern was observed for the trends according to the Brazilian States.

## INTRODUCTION

Prostate cancer is the second most commonly diagnosed cancer in men worldwide, with lung cancer being the first^1^. There were 1.4 million new cases of this neoplasm in the world in 2020, with an incidence rate of 30.7 cases per 100,000, and 7.7 deaths per 100,000 inhabitants/year, totaling 375,000 deaths on a global scale. In Brazil alone, there were more than 18,000 deaths caused by the disease in 2020^2^. 

Age and family history are the main risk factors for prostate cancer. Other factors may include excessive consumption of lipids, as well as the consumption of alcohol and smoking, although there is no consensus in literature^1^ ^,^ ^3^ ^-^ ^5^. Prostate cancer, in its early stages tends to be asymptomatic and has slow progress. In its advanced stages, it may manifest itself with symptoms in the lower urinary tract (LUT), microscopic hematuria, erectile dysfunction or nocturia, although those symptoms might occur due to benignant concomitant conditions or not^1^ ^,^ ^6^.

Population screening of prostate cancer is still controversial^7^. Its defenders base themselves on studies which show a relative reduction in specific cancer mortality of up to 9%, and explain this change with the introduction of prostate-specific antigen (PSA) monitoring^8^ ^,^ ^9^. Opponents base their opinions on systematic revisions, which show minimum or no impact on mortality and suggest that the risks and dangers of over-diagnosing and over-treatment outweigh the supposed modest benefits^10^.

The Brazilian Health Ministry and the National Cancer Institute do not recommend population screening for prostate cancer. Early detection should be performed for men who show symptoms related to the urinary tract or family history, and the risks inherent to the procedures should be discussed with the patient^11^ ^,^ ^12^.

In Brazil, an analysis of the mortality trend of prostate cancer, from 1980 to 2010, using data from the Brazilian Mortality Information System (SIM, in Portuguese), demonstrated an increase in mortality for the male Brazilian population of over 40 years of age in all regions of the country. An increase of 7.7% in deaths was reported after the redistribution of the poorly defined causes of death^13^. Further analysis with data up to 2014, and using a model of age-period-cohort, demonstrated an increase in mortality of men of 50 years of age and above over the last 30 years. Regional differences were identified, with a stable trend since 2004 in the South, Southeast, and Midwest regions, and an increase since 2000 in the North and Northeast regions. These trends might be related to access to health services for diagnosis and treatment. The significant effect of age was attributed mainly to population aging^3^. However, an analysis from the previous trend, from 1996 to 2010, had projected a reduction in prostate cancer mortality for Brazil as a whole^14^.

Some of the diverging results from previous studies^3^ ^,^ ^13^ ^,^ ^14^ might be due to methodological differences and problems with the quality of the records of deaths according to the period and the place studied. The present study sought to understand the phenomenon of prostate cancer mortality in Brazil. With the availability of estimates from corrected data from the Global Burden of Diseases (GBD) study, this study seeks to analyze the trend of prostate cancer mortality in Brazil and its States, in men of 40 years of age and above. 

## METHODS

An ecological study was conducted, which considered all deaths by prostate cancer that occurred among males of 40 years of age or above in Brazil, from 1990 to 2019. Data corrected and estimated by the GBD was used^15^ ^-^ ^17^. GBD estimates of mortality used a multiple approach, mainly considering vital records (data from the Mortality Information System - SIM, in Brazil) and cancer registries^18^ ^,^ ^19^. The data reported were mapped to a list of underlying causes in the GBD causes of death hierarchy^18^. Uninformative cause of death codes (the "garbage codes") are redistributed among appropriate underlying causes of death, as previously described^17^ ^,^ ^19^. Data on death were included in cancer-specific Cause of Death Ensemble models (CODEm) and were adjusted to independently modeled all-cause mortality (CodCorrect)^18^. This study defined deaths by prostate cancer by all records of deaths, which informed the basic cause of pathology from chapter 2, Group C61 - Malignant Prostate Neoplasm according to the International Statistical Classification of Diseases and Related Health Problems (ICD)-10th Revision. Data about deaths was collected according to the year and the area considered, from the population of 40 years of age or above; specific rates were calculated for different age groups (40-49, 50-59, 60-69, 70-79, and 80+). Population data were obtained by GBD from the Brazilian Institute of Geography and Statistics (IBGE). Overall mortality was standardized by the direct method, by the standard global population provided by the GBD study^20^. The crude and Age Standardized Mortality Rates (ASMR) were calculated per 100,000 inhabitants. Data was calculated for Brazil and its States.

The average annual percent change (AAPC) was calculated to identify trends of mortality, with a 95% confidence interval (95% CI) and a significance level of 5%. The AAPC is the weighted average of the angular coefficients of the line of regression, with equal weight for the length of each segment in the entire interval. An increase or decrease in the trend is significant when different from zero (p<0.05) and stable when equal to zero (p> 0.05). The trend analysis was performed by linear regression, using the Joinpoint Regression Program, version 4.8.0.1^21^. The maps to represent trends for Brazil and its states were produced by QGIS 3.12.

This study respected the ethical precepts for research and the specific Brazilian resolutions. It should be highlighted that the data was used in an aggregate format, without the identification of or harm to the individuals who participated in this study. The GBD study conforms to the guidelines for the reporting of precise and transparent health information. 

## RESULTS

**FIGURE 1:**
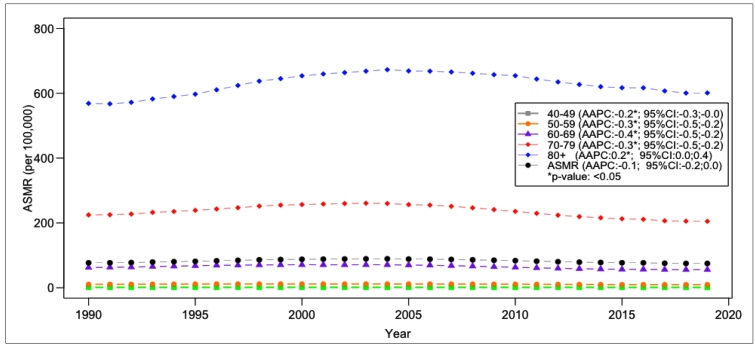
Prostate Cancer Mortality Rate, according to age groups, Brazil, 1990-2019. **AAPC:** Average Annual Percentage Change; **ASMR:** Age Standardized Mortality Rate. *value of p <0.05

**FIGURE 2:**
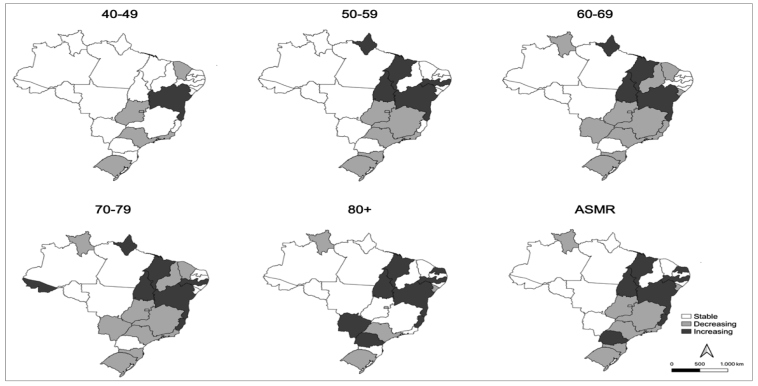
Trend of prostate cancer mortality, according to age group, for Brazil and its states, 1990-2019. **ASMR:** Age Standardized Mortality Rate.

For Brazil, the age-standardized rates went from 76.89 in 1990 to 74.96 deaths per 100,000 inhabitants, of 40 years of age or above in 2019, with a stable tendency in the studied time interval (Figure 1-2**,** Table 1 **,** Supplementary Table 1). 

The trend analysis identified that the state of Bahia showed the highest increase in mortality in the period (1.2 percentage point per year [p.p./year]), followed by Maranhão (1.0 p.p./year) and Pernambuco (0.9 p.p./year). Espírito Santo, Paraná, Rio Grande do Norte, and Tocantins showed an increasing trend but in lower proportion. A significant decrease in prostate cancer mortality was observed in the Federal District, Goiás, Minas Gerais, Rio de Janeiro, Rio Grande do Sul, Roraima, Santa Catarina, São Paulo, and Sergipe. The remaining states presented a stable tendency during the period, as did Brazil as a whole (Table 1, Figure 2 **,** Supplementary Table 1). 

**TABLE 1:** Standardized rate and average annual percentage of change (AAPC) of prostate cancer mortality in men ≥40 years of age, according State and year, 1990-2019.

States	ASMR			
	1990	2019	AAPC	95% CI
Acre	84.05	82.94	-0.1	(-0.4;0.1)
Alagoas	66.08	69.09	0.1	(-0.1;0.3)
Amapá	71.47	76.34	0.2	(-0.3;0.7)
Amazonas	71.20	76.55	0.2	(-0.2;0.7)
Bahia	72.54	100.18	1.2*	(1.0;1.4)
Ceará	80.98	78.87	-0.1	(-0.5;0.3)
Distrito Federal	121.79	84.94	-1.3*	(-1.7;-0.8)
Espírito Santo	64.82	77.06	0.6*	(0.4;0.7)
Goiás	82.33	72.07	-0.4*	(-0.7;-0.2)
Maranhão	71.84	93.96	1.0*	(0.2;1.7)
Mato Grosso	79.72	73.43	-0.4	(-0.8;0.1)
Mato Grosso do Sul	73.50	72.13	-0.1	(-0.2;0.1)
Minas Gerais	72.54	65.69	-0.4*	(-0.6;-0.2)
Pará	64.30	66.20	0.1	(-0.1;0.4)
Paraíba	73.31	72.16	0.0	(-0.7;0.7)
Paraná	69.65	77.53	0.4*	(0.1;0.6)
Pernambuco	68.73	86.12	0.9*	(0.6;1.1)
Piauí	73.74	65.25	-0.4	(-1.0;0.1)
Rio de Janeiro	83.21	78.16	-0.2*	(-0.4;-0.0)
Rio Grande do Norte	66.07	74.87	0.4*	(0.2;0.6)
Rio Grande do Sul	90.44	76.72	-0.6*	(-0.7;-0.4)
Rondônia	90.59	81.52	-0.2	(-0.8;0.4)
Roraima	119.24	90.11	-1.0*	(-1.2;-0.7)
Santa Catarina	77.78	69.83	-0.4*	(-0.6;-0.1)
São Paulo	81.23	66.80	-0.7*	(-0.8;-0.5)
Sergipe	98.69	82.50	-0.6*	(-1.1;-0.2)
Tocantins	78.15	97.66	0.8*	(0.0;1.6)
Brazil	76.89	74.96	-0.1	(-0.2;0.0)

**ASMR:** Age Standardized Mortality Rate according to age group distribution of the global population, shown in number of deaths per 100,000 inhabitants.**CI:** Confidence Interval. *p-value <0.05

Among the studied age groups, for Brazil, the mortality rates showed a tendency of decrease between 40 and 79 years of age, and an increase for 80 years of age and older (0.2 p.p./year) (Table 2). No pattern in the trends was found for states according to age groups (Table 2 **,** Figure 2,  Supplementary Tables 2-6). The increasing trend in Bahia and the decreasing trend in Rio Grande do Sul and São Paulo for men of 40 years of age and above stand out. 

**TABLE 2:** Prostate Cancer mortality rate (per 100,000 inhabitants) and average annual percentage of change (AAPC), according to age group, for Brazil and for its states, 1990 and 2019.

States	40-49 years of age			50-59 years of age			60-69 years of age			70-79 years of age			≥80 years of age		
	1990	2019	AAPC	1990	2019	AAPC	1990	2019	AAPC	1990	2019	AAPC	1990	2019	AAPC
Acre	1.11	1.15	0.0	8.64	9.24	0.2	50.53	52.32	0.1	189.51	209.65	0.4*	798.72	734.27	-0.3
Alagoas	1.25	1.28	0.1	9.10	9.64	0.1	54.33	56,.34	0.1	199.93	192.25	-0.1	473.03	527.11	0.3
Amapá	0.91	1.07	0.5	7.70	8.85	0.5*	47.51	52.52	0.3*	177.67	208.53	0.5*	632.28	631.08	-0.1
Amazonas	1.07	1.15	0.2	8.94	9.21	0.2	55.55	54.93	-0.1	197.77	221.93	0.4	557.75	599.49	0.3
Bahia	1.31	1.50	0.5*	10.37	13.79	1.0*	61.88	82.82	1.0*	214.58	284.50	1.0*	519.81	752.71	1.3*
Ceará	1.33	1.23	-0.3*	10.44	9.87	-0.2	59.82	53.38	-0.4*	240.18	207.52	-0.5*	614.75	665.78	0.3
Distrito Federal	1.24	0.94	-1.0*	10.46	7.68	-1.1*	67.96	50.00	-1.0*	281.24	206.39	-1.0*	1173.27	788.52	-1.3*
Espírito Santo	1.05	1.11	0.2	8.75	9.27	0.2	53.85	50.66	-0.3*	184.54	207.43	0.4*	485.67	648.74	1.0*
Goiás	1.50	1.10	-1.0*	12.12	8.81	-1.1*	69.87	54.31	-0.9*	240.80	199.01	-0.7*	594.57	574.04	-0.1
Maranhão	1.31	1.25	-0.2	9.84	11.17	0.5*	57.37	66.68	0.5*	195.24	234.18	0.7*	561.63	811.37	1.5*
Mato Grosso	1.21	1.18	-0.0	9.80	9.46	-0.1	57.68	53.03	-0.3	242.55	210.26	-0.5	600.44	575.62	-0.2
Mato Grosso do Sul	1.22	1.15	-0.2	9.76	9.04	-0.3	60.63	50.47	-0.6*	216.45	194.08	-0.4*	539.15	595.28	0.4*
Minas Gerais	1.19	1.17	-0.0	10.52	9.45	-0.4*	57.70	50.68	-0.5*	212.38	181.80	-0.5*	537.46	511.35	-0.2
Pará	1.00	1.04	0.1	8.82	8.39	-0.2	50.88	50.88	0.0	193.47	188.16	-0.1	470.04	512.50	0.3
Paraíba	1.24	1.27	0.1	9.86	10.37	0.2	57.55	57.37	-0.0	214.77	194.96	-0.4	548.65	565.24	0.1
Paraná	1.03	1.06	0.1	9.22	9.18	0.0	56.64	54.47	-0.1	196.60	213.37	0.3	530.30	633.58	0.6*
Pernambuco	1.22	1.35	0.3	9.43	11.41	0.6*	59.94	62.45	0.2	200.10	241.07	0.7*	496.23	683.47	1.1*
Piauí	1.15	1.09	-0.2	9.17	8.67	-0.2	58.43	52.28	-0.4*	216.55	174.78	-0.8*	552.93	516.21	-0.3
Rio de Janeiro	1.40	1.15	-0.7*	12.30	10.63	-0.5*	70.70	60.47	-0.5*	250.56	217.28	-0.5*	587.64	610.12	0.2
Rio Grande do Norte	1.12	1.16	0.2	8.07	9.13	0.5	52.37	52.55	-0.0	189.85	202.05	0.2	503.30	617.64	0.7*
Rio Grande do Sul	1.28	1.07	-0.6*	12.44	8.77	-1.2*	70.42	55.46	-0.8*	265.72	210.49	-0.8*	677.42	624.47	-0.3*
Rondônia	1.32	1.25	-0.1	9.89	9.49	-0.1	57.39	60.94	0.2	241.24	226.78	-0.1	778.89	649.77	-0.4
Roraima	1.47	1.26	-0.6	11.22	11.12	-0.0	82.61	63.38	-0.9*	306.87	225.94	-1.0*	1031.91	775.39	-1.0*
Santa Catarina	1.03	0.97	-0.2	9.61	8.24	-0.6*	61.42	47.42	-0.9*	216.72	190.22	-0.4*	607.15	579.71	-0.2
São Paulo	1.21	1.03	-0.5*	11.03	8.57	-0.9*	65.39	50.40	-0.9*	239.00	179.37	-1.0*	601.08	539.55	-0.4*
Sergipe	1.45	1.40	-0.1	11.83	11.21	-0.2	69.37	60.85	-0.4*	245.49	236.88	-0.1	854.10	638.43	-1.3*
Tocantins	1.18	1.32	0.4	8.89	10.98	0.7*	56.28	65.46	0.5*	193.04	234.92	0.7*	676.53	874.43	0.9*
Brazil	1.23	1.15	-0.2*	10.62	9.62	-0.3*	62.45	55.75	-0.4*	224.71	204.98	-0.3*	568.71	601.06	0.2*

*p-value <0.05.

## DISCUSSION

This study evaluates the trends of prostate cancer mortality in Brazil and its states, from 1990 to 2019. The results show that mortality, corrected by the GBD, was stable in Brazil. However, according to the Brazilian states, disparities were observed. 

Although stability can be found in the mortality rates in most of the states of Brazil, with no specific regional pattern, the corrected mortality for the initial year (1990) and final year (2019) in the series make the states of Bahia, Maranhão, and Tocantins (from the Northeast and North regions of Brazil) stand out due to the highest rates identified at the end of the series, and the highest increase during the period. By contrast, of the nine states with a trend leaning toward a reduction in mortality, seven are in the Midwest, Southeast, and South regions of the country. Such diversities match the estimates projected for 2025, with data from 2010^14^, suggesting that the highest mortality in the less developed regions was related to a limited access to diagnosis and treatment, as well as to the lower quality of health services and information. Such inequalities were also indicated in other national studies^3^ ^,^ ^22^, including differences between the capitals and the more outlying regions of the country^23^.

One can consider that the improvement in life expectancy in some of the Brazilian states, such as Bahia, may not be accompanied by healthier habits, nor access to health services and preventive education. The population, urbanized and older, fall ill more often and, with a more delayed diagnosis, ends up dying because of the disease. States, such as Rio Grande do Sul and São Paulo, where conditions of access to health and educational and income standards are higher, when compared to states from the North and Northeastern regions of the country, demonstrate a trend toward a decrease in mortality for all age groups^24^.

When data is separated by age group, mortality in Brazil tends to be higher for those people of 80 years of age and above, and stable before that age, which is consistent with findings from the literature, which describes prostate cancer as a disease of elderly men^25^. However, this goes against what has been happening in other countries, such as in some European countries^26^ ^,^ ^27^ and in North America^28^, which show a decrease in mortality in the last years of analysis; in China as well, where mortality has decreased between 1990 and 2017 for people 40 years of age and above^29^. Among the South American countries (Argentina, Brazil, Chile, Colombia, Cuba, Mexico, and Venezuela), a tendency of decrease in prostate cancer mortality of men was estimated at every age, between 2012 and 2017^30^. Regardless of the identified trends, a study based on the GBD, which evaluated mortality by cancer around the world, indicated that prostate cancer was the most prevalent cancer in 114 countries in 2017, and the main cause of death by cancer, for men in 56 countries^18^.

Other factors, such as the successful screening of the studied neoplasm, may have contributed to improved records of mortality^22^. International studies indicate that the reduction in prostate cancer mortality is due to increased screening^27^ ^,^ ^28^ ^,^ ^31^, as well as to improvements in the treatments for the disease^32^. Measures of population screening are not recommended in Brazil because of the limited evidence of cost-benefit, due to the possibility of overdiagnosis^23^ ^,^ ^33^ ^,^ ^34^. Therefore, the diagnostic exams are performed on demand, and it is expected that they will be more frequent in places where there is more interest and access to health services. It is well-known that the spontaneous interest for health services on the part of the male population is low^35^. Studies indicate that in some parts of the country, less than 50% of men seek medical attention. When it does happen, the interest is mainly due to the presence of already evident symptoms or due to an urgent need^36^ ^,^ ^37^. Nevertheless, tendency studies indicate an increase in the number of men who sought out health services from 2008 to 2013 in Brazil, especially in the Southeast and South regions^38^. This may have contributed to a reduction in mortality verified in some states in those regions. The Ministry of Health’s creation of the national policy of overall attention to the health of men, formulated in 2009, aims to provide more attention and health education to the male population at the primary level of attention to health, which may improve inclusion and, consequently, impact mortality due to prostate cancer^39^.

The effect of the differences in lifestyles among countries should also be considered, and in the trend analysis, the changes in terms of exposure to risk factors related to susceptibility and cancer mortality, although there is no consensus in literature on this issue^1^ ^,^ ^33^. Although there has been a reduction in smoking and an increase in physical activity and consumption of fruits and vegetables among Brazilian men, up to 2019, the prevalence of excessive weight and obesity was still increasing^40^, which may have had implications in the development of this cancer. Smoking is one of the main risk factors associated with prostate cancer and it has been connected to the high incidence of prostate cancer^4^ ^,^ ^41^ ^,^ ^42^.

Besides being one of the most common risk factors for the development of cancer, the habit of smoking at the time of diagnosis and treatment of the disease demonstrated, in a meta-analysis, a negative impact on the patient's prognosis, which is associated with a lower survival rate^43^. Regardless of the decreasing trend verified in Brazil^40^, it is estimated that 15.9% of Brazilian men are smokers, especially in the Southeast and South regions^44^, which is an important factor to be addressed in the prevention of prostate cancer^45^.

The identification of a stable trend in prostate cancer mortality, differently from the findings of different studies which show an increase in mortality in Brazil and its regions, may be related to differences in the methodologies used in the publications^3^ ^,^ ^46^ ^,^ ^47^. The evaluation of causes of death and trends from 1990 to 2019 for Brazil may be affected by the quality of data and by changes in the information systems in the period of each study. Therefore, the use of secondary data, which reports deaths without an adequate record of causes, influenced by the lack of a precise diagnosis, may be considered an overall limitation in the studies of mortality. The comparisons between the estimates from different studies must be performed carefully, considering such specificities. 

The use of corrected data and the redistribution of *garbage* codes^48^, obtained from the GBD study, bring the data closer to reality. This happens because the GBD study tries to minimize the lack of secondary data and its low quality by using diverse sources, such as verbal records of cancer and autopsy. It also adjusts the estimates, with correction for the poorly defined causes of death. The present study highlights that, since it uses a globally standardized methodology, the study has the potential of allowing comparisons of mortality between different Brazilian states and regions, as well as between Brazil and other countries. 

Mortality by prostate cancer in men 40 years of age and above, in Brazil, has remained stable in the period of 1990 to 2019, with an increase in the population of 80 years of age and above, and with regional differences, which proved to be more prevalent in states from the Northeast and North regions. The heterogeneity found in this study may be a reflection of economic factors, of education, and of access to health care, for both early diagnosis and adequate treatment. 
