# Supplementary material for: Prostate cancer mortality in Brazil 1990-2019: geographical distribution and trends
Source: Rev Soc Bras Med Trop. 2022 Jan 28;55(Suppl 1):e0277-2021. doi: 10.1590/0037-8682-0277-2021 (PMC9020381; doi:10.1590/0037-8682-0277-2021)
Supplement: Supplementary file 1 [file 1678-9849-rsbmt-55-s01-e0277-2021-supp1.pdf]

Supplementary Table 1. Standardized rate of prostate câncer mortality in men ≥40 years of age, according State and year, 1990-2019.

Brazilian States	Year																														AAPC	95%CI
	1990	1991	1992	1993	1994	1995	1996	1997	1998	1999	2000	2001	2002	2003	2004	2005	2006	2007	2008	2009	2010	2011	2012	2013	2014	2015	2016	2017	2018	2019		
Acre	84.05	84.65	84.92	82.43	80.11	76.25	79.25	80.97	78.55	76.65	78.83	79.76	79.73	79.30	83.06	83.35	85.95	85.26	84.47	84.09	85.94	86.78	87.76	86.29	85.08	83.74	82.44	81.98	82.82	82.94	-0.1	(-0.4;0.1)
Alagoas	66.08	65.65	65.43	65.10	64.29	63.49	62.56	62.07	62.08	64.32	64.41	64.17	65.52	66.79	68.39	69.09	69.60	70.58	70.41	70.65	70.29	69.66	71.11	72.40	72.10	71.27	69.34	68.87	69.22	69.09	0.1	(-0.1;0.3)
Amapá	71.47	74.56	74.37	75.11	77.13	78.36	78.19	76.17	75.96	75.89	76.11	76.21	78.61	77.23	75.34	73.47	73.83	74.00	75.77	74.68	73.76	73.46	75.52	77.31	78.23	78.65	78.86	76.89	76.64	76.34	0.2	(-0.3;0.7)
Amazonas	71.20	69.38	70.25	70.38	71.12	70.72	68.55	68.65	71.41	75.72	78.75	79.38	79.74	80.13	80.04	80.96	79.35	78.57	78.00	75.75	74.97	74.42	74.30	74.67	75.53	77.12	77.60	76.59	75.74	76.55	0.2	(-0.2;0.7)
Bahia	72.54	73.67	75.34	77.50	78.12	80.35	81.97	84.45	86.77	89.21	91.62	93.71	95.80	96.99	99.62	102.52	105.71	107.96	109.17	108.93	107.38	105.22	105.61	104.61	103.39	103.17	103.27	102.67	101.01	100.18	1.2*	(1.0;1.4)
Ceará	80.98	82.12	82.64	84.16	86.80	92.09	93.29	93.62	90.59	88.33	88.40	85.99	86.12	87.86	88.58	88.49	89.01	88.33	87.38	85.71	83.65	81.33	80.40	79.70	80.01	80.63	81.32	80.74	78.82	78.87	-0.1	(-0.5;0.3)
Distrito Federal	121.79	119.25	113.82	108.55	107.65	105.04	105.97	108.65	105.74	102.74	100.21	103.41	104.75	103.28	102.00	100.65	100.47	101.14	103.43	104.52	104.85	102.14	97.93	94.07	92.25	88.39	85.56	82.73	84.76	84.94	-1.3*	(-1.7;-0.8)
Espírito Santo	64.82	65.10	65.17	65.74	65.76	63.95	65.58	64.38	67.13	66.19	67.05	67.27	67.14	68.47	70.79	70.26	71.14	71.70	72.17	72.96	74.29	75.03	75.61	75.16	76.85	77.91	78.46	78.10	76.96	77.06	0.6*	(0.4;0.7)
Goiás	82.33	82.04	82.56	83.90	84.72	87.44	90.86	93.47	95.12	96.04	95.75	96.22	94.50	93.71	94.15	93.55	92.90	90.90	87.60	86.03	85.27	82.32	79.73	77.15	76.01	74.82	73.06	70.98	71.79	72.07	-0.4*	(-0.7;-0.2)
Maranhão	71.84	71.48	72.41	71.61	69.55	69.09	72.25	75.83	74.13	72.17	69.85	66.27	63.03	65.66	59.31	57.26	57.45	60.44	63.97	66.76	73.54	95.80	95.28	95.40	95.96	94.88	94.40	94.35	94.07	93.96	1.0*	(0.2;1.7)
Mato Grosso	79.72	80.68	79.08	79.54	77.42	79.62	80.89	82.97	85.41	91.06	94.43	95.33	95.45	97.64	98.40	98.78	95.97	93.59	93.00	89.61	88.05	84.61	81.76	79.88	79.58	77.95	76.96	73.99	73.28	73.43	-0.4	(-0.8;0.1)
Mato Grosso do Sul	73.50	73.61	73.93	76.06	78.54	79.39	81.84	82.68	84.89	87.03	86.62	84.87	85.10	87.93	88.50	87.07	86.93	85.55	85.60	83.28	82.51	79.85	78.86	77.73	76.10	75.47	75.29	72.38	71.84	72.13	-0.1	(-0.2;0.1)
Minas Gerais	72.54	72.08	72.69	73.44	73.92	74.00	75.13	76.84	79.94	81.72	85.19	89.75	92.49	90.39	91.03	92.28	91.47	89.47	87.14	84.69	82.17	77.89	75.28	72.44	69.82	68.72	67.58	65.58	65.38	65.69	-0.4*	(-0.6;-0.2)
Pará	64.30	63.55	63.20	63.35	63.37	62.74	63.76	64.30	64.67	66.65	70.05	69.63	71.45	74.61	75.18	72.54	72.39	72.62	72.91	73.88	74.91	71.43	69.48	69.62	69.10	68.11	68.25	67.28	66.43	66.20	0.1	(-0.1;0.4)
Paraíba	73.31	73.96	73.52	72.60	76.04	80.07	81.43	82.59	82.85	84.19	84.73	80.80	78.87	79.15	83.88	86.23	86.68	86.76	89.42	85.94	84.52	82.18	77.91	75.26	74.74	74.64	74.04	72.99	71.86	72.16	0.0	(-0.7;0.7)
Paraná	69.65	69.53	71.17	73.78	74.81	76.13	79.65	81.83	84.65	85.65	86.63	87.72	88.14	89.04	89.85	87.30	86.79	85.62	84.98	84.84	84.52	83.21	82.59	81.72	80.25	79.63	78.85	77.79	77.38	77.53	0.4*	(0.1;0.6)
Pernambuco	68.73	68.13	67.84	70.86	71.20	72.17	74.28	76.87	79.65	80.83	82.17	82.73	85.92	88.87	90.78	90.37	90.64	90.75	91.07	90.65	90.13	90.16	88.81	88.38	88.42	89.28	89.63	89.38	87.03	86.12	0.9*	(0.6;1.1)
Piauí	73.74	71.62	72.53	71.85	69.75	68.10	68.68	74.98	78.19	81.54	87.52	90.02	91.66	89.25	84.25	82.60	80.42	74.65	71.69	72.36	75.59	72.00	71.90	69.24	66.26	65.05	64.86	65.24	64.75	65.25	-0.4	(-1.0;0.1)
Rio de Janeiro	83.21	84.12	85.18	88.02	90.08	90.39	91.24	91.23	93.03	93.37	92.88	94.40	94.42	94.27	93.97	92.07	91.26	91.18	89.24	87.02	84.95	81.91	80.27	79.79	78.02	77.47	79.24	78.33	78.07	78.16	-0.2*	(-0.4;-0.0)
Rio Grande do Norte	66.07	67.78	68.47	69.60	71.13	70.43	70.55	70.27	71.90	72.21	73.31	76.50	78.93	81.69	82.67	85.07	88.46	88.81	84.95	80.94	78.32	76.05	75.00	74.31	74.82	75.13	75.57	75.14	74.73	74.87	0.4*	(0.2;0.6)
Rio Grande do Sul	90.44	91.09	92.80	95.50	96.18	96.91	98.38	98.63	100.73	100.45	99.29	98.51	97.45	97.04	97.06	94.56	93.24	91.87	89.23	87.65	86.45	83.10	81.62	80.47	79.05	77.07	77.41	75.91	76.37	76.72	-0.6*	(-0.7;-0.4)
Rondônia	90.59	72.40	68.66	76.04	91.74	94.96	86.94	84.49	82.31	80.12	81.34	79.82	76.69	78.95	78.78	78.61	78.15	76.07	76.41	75.73	76.13	76.47	76.47	76.85	80.01	81.03	80.36	80.61	81.27	81.52	-0.2	(-0.8;0.4)
Roraima	119.24	121.01	118.81	114.25	114.64	114.52	112.56	110.87	110.68	102.61	103.44	103.05	102.85	103.11	101.42	99.47	99.14	99.24	95.32	94.41	94.70	94.25	94.37	92.02	88.89	87.81	87.42	88.73	89.70	90.11	-1.0*	(-1.2;-0.7)
Santa Catarina	77.78	79.13	80.02	80.59	82.15	82.89	84.12	85.32	88.14	88.40	89.16	90.26	89.61	89.42	89.80	87.62	85.98	84.27	82.17	81.17	79.79	76.36	73.18	71.47	70.64	69.97	69.56	68.40	69.20	69.83	-0.4*	(-0.6;-0.1)
São Paulo	81.23	80.72	81.54	83.92	85.93	87.71	90.31	91.83	93.45	94.26	93.77	93.24	92.48	92.16	90.95	87.98	86.23	83.78	81.91	80.21	77.72	74.91	72.99	71.42	70.20	69.34	69.10	67.15	66.74	66.80	-0.7*	(-0.8;-0.5)
Sergipe	98.69	97.68	96.93	95.32	93.27	92.30	88.78	88.92	92.81	94.52	96.22	96.99	101.78	104.83	105.89	103.10	103.17	106.11	103.91	99.31	96.79	93.26	93.43	92.26	88.72	83.86	82.46	81.63	82.13	82.50	-0.6*	(-1.1;-0.2)
Tocantins	78.15	73.96	72.59	70.99	68.30	68.20	68.96	69.37	68.74	69.11	67.60	68.36	69.23	71.66	74.02	79.41	81.74	77.45	75.72	79.20	85.80	87.64	90.60	99.10	107.85	106.51	105.10	102.20	98.91	97.66	0.8*	(0.0;1.6)
**Brazil**	76.89	76.95	77.67	79.30	80.38	81.55	83.17	84.56	86.16	87.02	87.80	88.30	88.72	89.15	89.35	88.51	88.16	87.27	86.15	84.82	83.62	81.87	80.30	79.01	77.88	77.11	76.89	75.52	74.96	74.96	-0.1	(-0.2;0.0)

AAPC = annual average percent change
95% CI= 95% Confidence Interval
*Statistical significance at level of 5%
