# Supplementary material for: Prostate cancer mortality in Brazil 1990-2019: geographical distribution and trends
Source: Rev Soc Bras Med Trop. 2022 Jan 28;55(Suppl 1):e0277-2021. doi: 10.1590/0037-8682-0277-2021 (PMC9020381; doi:10.1590/0037-8682-0277-2021)
Supplement: Supplementary file 2 [file 1678-9849-rsbmt-55-s01-e0277-2021-supp2.pdf]

Supplementary Table 2. Standardized rate of prostate câncer mortality in men of 40-49 years of age, according State and year, 1990-2019.

Brazilian States	Year																														AAPC	95%CI
	1990	1991	1992	1993	1994	1995	1996	1997	1998	1999	2000	2001	2002	2003	2004	2005	2006	2007	2008	2009	2010	2011	2012	2013	2014	2015	2016	2017	2018	2019		
Acre	84.05	84.65	84.92	82.43	80.11	76.25	79.25	80.97	78.55	76.65	78.83	79.76	79.73	79.30	83.06	83.35	85.95	85.26	84.47	84.09	85.94	86.78	87.76	86.29	85.08	83.74	82.44	81.98	82.82	82.94	-0.1	(-0.4;0.1)
Alagoas	66.08	65.65	65.43	65.10	64.29	63.49	62.56	62.07	62.08	64.32	64.41	64.17	65.52	66.79	68.39	69.09	69.60	70.58	70.41	70.65	70.29	69.66	71.11	72.40	72.10	71.27	69.34	68.87	69.22	69.09	0.1	(-0.1;0.3)
Amapá	71.47	74.56	74.37	75.11	77.13	78.36	78.19	76.17	75.96	75.89	76.11	76.21	78.61	77.23	75.34	73.47	73.83	74.00	75.77	74.68	73.76	73.46	75.52	77.31	78.23	78.65	78.86	76.89	76.64	76.34	0.2	(-0.3;0.7)
Amazonas	71.20	69.38	70.25	70.38	71.12	70.72	68.55	68.65	71.41	75.72	78.75	79.38	79.74	80.13	80.04	80.96	79.35	78.57	78.00	75.75	74.97	74.42	74.30	74.67	75.53	77.12	77.60	76.59	75.74	76.55	0.2	(-0.2;0.7)
Bahia	72.54	73.67	75.34	77.50	78.12	80.35	81.97	84.45	86.77	89.21	91.62	93.71	95.80	96.99	99.62	102.52	105.71	107.96	109.17	108.93	107.38	105.22	105.61	104.61	103.39	103.17	103.27	102.67	101.01	100.18	1.2*	(1.0;1.4)
Ceará	80.98	82.12	82.64	84.16	86.80	92.09	93.29	93.62	90.59	88.33	88.40	85.99	86.12	87.86	88.58	88.49	89.01	88.33	87.38	85.71	83.65	81.33	80.40	79.70	80.01	80.63	81.32	80.74	78.82	78.87	-0.1	(-0.5;0.3)
Distrito Federal	121.79	119.25	113.82	108.55	107.65	105.04	105.97	108.65	105.74	102.74	100.21	103.41	104.75	103.28	102.00	100.65	100.47	101.14	103.43	104.52	104.85	102.14	97.93	94.07	92.25	88.39	85.56	82.73	84.76	84.94	-1.3*	(-1.7;-0.8)
Espírito Santo	64.82	65.10	65.17	65.74	65.76	63.95	65.58	64.38	67.13	66.19	67.05	67.27	67.14	68.47	70.79	70.26	71.14	71.70	72.17	72.96	74.29	75.03	75.61	75.16	76.85	77.91	78.46	78.10	76.96	77.06	0.6*	(0.4;0.7)
Goiás	82.33	82.04	82.56	83.90	84.72	87.44	90.86	93.47	95.12	96.04	95.75	96.22	94.50	93.71	94.15	93.55	92.90	90.90	87.60	86.03	85.27	82.32	79.73	77.15	76.01	74.82	73.06	70.98	71.79	72.07	-0.4*	(-0.7;-0.2)
Maranhão	71.84	71.48	72.41	71.61	69.55	69.09	72.25	75.83	74.13	72.17	69.85	66.27	63.03	65.66	59.31	57.26	57.45	60.44	63.97	66.76	73.54	95.80	95.28	95.40	95.96	94.88	94.40	94.35	94.07	93.96	1.0*	(0.2;1.7)
Mato Grosso	79.72	80.68	79.08	79.54	77.42	79.62	80.89	82.97	85.41	91.06	94.43	95.33	95.45	97.64	98.40	98.78	95.97	93.59	93.00	89.61	88.05	84.61	81.76	79.88	79.58	77.95	76.96	73.99	73.28	73.43	-0.4	(-0.8;0.1)
Mato Grosso do Sul	73.50	73.61	73.93	76.06	78.54	79.39	81.84	82.68	84.89	87.03	86.62	84.87	85.10	87.93	88.50	87.07	86.93	85.55	85.60	83.28	82.51	79.85	78.86	77.73	76.10	75.47	75.29	72.38	71.84	72.13	-0.1	(-0.2;0.1)
Minas Gerais	72.54	72.08	72.69	73.44	73.92	74.00	75.13	76.84	79.94	81.72	85.19	89.75	92.49	90.39	91.03	92.28	91.47	89.47	87.14	84.69	82.17	77.89	75.28	72.44	69.82	68.72	67.58	65.58	65.38	65.69	-0.4*	(-0.6;-0.2)
Pará	64.30	63.55	63.20	63.35	63.37	62.74	63.76	64.30	64.67	66.65	70.05	69.63	71.45	74.61	75.18	72.54	72.39	72.62	72.91	73.88	74.91	71.43	69.48	69.62	69.10	68.11	68.25	67.28	66.43	66.20	0.1	(-0.1;0.4)
Paraíba	73.31	73.96	73.52	72.60	76.04	80.07	81.43	82.59	82.85	84.19	84.73	80.80	78.87	79.15	83.88	86.23	86.68	86.76	89.42	85.94	84.52	82.18	77.91	75.26	74.74	74.64	74.04	72.99	71.86	72.16	0.0	(-0.7;0.7)
Paraná	69.65	69.53	71.17	73.78	74.81	76.13	79.65	81.83	84.65	85.65	86.63	87.72	88.14	89.04	89.85	87.30	86.79	85.62	84.98	84.84	84.52	83.21	82.59	81.72	80.25	79.63	78.85	77.79	77.38	77.53	0.4*	(0.1;0.6)
Pernambuco	68.73	68.13	67.84	70.86	71.20	72.17	74.28	76.87	79.65	80.83	82.17	82.73	85.92	88.87	90.78	90.37	90.64	90.75	91.07	90.65	90.13	90.16	88.81	88.38	88.42	89.28	89.63	89.38	87.03	86.12	0.9*	(0.6;1.1)
Piauí	73.74	71.62	72.53	71.85	69.75	68.10	68.68	74.98	78.19	81.54	87.52	90.02	91.66	89.25	84.25	82.60	80.42	74.65	71.69	72.36	75.59	72.00	71.90	69.24	66.26	65.05	64.86	65.24	64.75	65.25	-0.4	(-1.0;0.1)
Rio de Janeiro	83.21	84.12	85.18	88.02	90.08	90.39	91.24	91.23	93.03	93.37	92.88	94.40	94.42	94.27	93.97	92.07	91.26	91.18	89.24	87.02	84.95	81.91	80.27	79.79	78.02	77.47	79.24	78.33	78.07	78.16	-0.2*	(-0.4;-0.0)
Rio Grande do Norte	66.07	67.78	68.47	69.60	71.13	70.43	70.55	70.27	71.90	72.21	73.31	76.50	78.93	81.69	82.67	85.07	88.46	88.81	84.95	80.94	78.32	76.05	75.00	74.31	74.82	75.13	75.57	75.14	74.73	74.87	0.4*	(0.2;0.6)
Rio Grande do Sul	90.44	91.09	92.80	95.50	96.18	96.91	98.38	98.63	100.73	100.45	99.29	98.51	97.45	97.04	97.06	94.56	93.24	91.87	89.23	87.65	86.45	83.10	81.62	80.47	79.05	77.07	77.41	75.91	76.37	76.72	-0.6*	(-0.7;-0.4)
Rondônia	90.59	72.40	68.66	76.04	91.74	94.96	86.94	84.49	82.31	80.12	81.34	79.82	76.69	78.95	78.78	78.61	78.15	76.07	76.41	75.73	76.13	76.47	76.47	76.85	80.01	81.03	80.36	80.61	81.27	81.52	-0.2	(-0.8;0.4)
Roraima	119.24	121.01	118.81	114.25	114.64	114.52	112.56	110.87	110.68	102.61	103.44	103.05	102.85	103.11	101.42	99.47	99.14	99.24	95.32	94.41	94.70	94.25	94.37	92.02	88.89	87.81	87.42	88.73	89.70	90.11	-1.0*	(-1.2;-0.7)
Santa Catarina	77.78	79.13	80.02	80.59	82.15	82.89	84.12	85.32	88.14	88.40	89.16	90.26	89.61	89.42	89.80	87.62	85.98	84.27	82.17	81.17	79.79	76.36	73.18	71.47	70.64	69.97	69.56	68.40	69.20	69.83	-0.4*	(-0.6;-0.1)
São Paulo	81.23	80.72	81.54	83.92	85.93	87.71	90.31	91.83	93.45	94.26	93.77	93.24	92.48	92.16	90.95	87.98	86.23	83.78	81.91	80.21	77.72	74.91	72.99	71.42	70.20	69.34	69.10	67.15	66.74	66.80	-0.7*	(-0.8;-0.5)
Sergipe	98.69	97.68	96.93	95.32	93.27	92.30	88.78	88.92	92.81	94.52	96.22	96.99	101.78	104.83	105.89	103.10	103.17	106.11	103.91	99.31	96.79	93.26	93.43	92.26	88.72	83.86	82.46	81.63	82.13	82.50	-0.6*	(-1.1;-0.2)
Tocantins	78.15	73.96	72.59	70.99	68.30	68.20	68.96	69.37	68.74	69.11	67.60	68.36	69.23	71.66	74.02	79.41	81.74	77.45	75.72	79.20	85.80	87.64	90.60	99.10	107.85	106.51	105.10	102.20	98.91	97.66	0.8*	(0.0;1.6)
**Brazil**	76.89	76.95	77.67	79.30	80.38	81.55	83.17	84.56	86.16	87.02	87.80	88.30	88.72	89.15	89.35	88.51	88.16	87.27	86.15	84.82	83.62	81.87	80.30	79.01	77.88	77.11	76.89	75.52	74.96	74.96	-0.1	(-0.2;0.0)

AAPC = annual average percent change
95% CI= 95% Confidence Interval
*Statistical significance at level of 5%

Supplementary Table 3. Standardized rate of prostate câncer mortality in men of 50-59 years of age, according State and year, 1990-2019.

Brazilian States	Year																														AAPC	95%CI
	1990	1991	1992	1993	1994	1995	1996	1997	1998	1999	2000	2001	2002	2003	2004	2005	2006	2007	2008	2009	2010	2011	2012	2013	2014	2015	2016	2017	2018	2019		
Acre	8.64	8.95	9.12	9.09	9.30	9.14	9.42	9.40	9.34	9.20	9.30	9.48	9.56	9.69	9.45	9.13	8.85	9.31	9.53	9.80	9.74	9.34	9.37	9.23	9.24	9.11	9.09	9.02	9.22	9.24	0.2	(-0.4;0.8)
Alagoas	9.10	9.17	9.20	9.14	9.09	9.06	9.00	9.09	9.12	8.88	9.32	9.31	9.70	9.72	9.90	9.78	9.82	9.76	9.82	9.90	10.05	10.01	10.10	10.03	9.87	9.92	9.61	9.44	9.57	9.64	0.1	(-0.2;0.4)
Amapá	7.70	7.77	8.06	8.47	8.68	8.77	8.87	8.91	8.81	8.85	8.97	9.16	9.15	9.17	8.78	8.34	8.15	8.25	8.41	8.49	8.55	8.64	8.87	8.93	9.01	9.05	8.92	8.71	8.82	8.85	0.5*	(0.1;0.8)
Amazonas	8.94	8.53	8.93	8.90	9.11	9.27	9.26	9.31	9.58	9.84	10.19	10.41	10.31	10.15	10.13	10.01	10.06	9.87	9.84	9.66	9.65	9.32	9.18	9.08	9.22	9.17	8.99	8.85	9.04	9.21	0.2	(-0.2;0.6)
Bahia	10.37	10.40	10.62	10.87	11.11	11.44	11.69	12.01	12.18	12.59	12.95	12.80	13.15	13.55	13.70	13.70	13.72	13.88	13.92	13.83	13.81	13.93	14.22	14.11	14.04	14.09	13.86	13.91	13.81	13.79	1.0*	(0.9;1.2)
Ceará	10.44	10.65	10.77	10.96	11.14	11.44	11.64	11.57	11.19	11.23	11.08	11.02	11.10	11.47	11.57	11.57	11.69	11.65	11.41	11.08	10.70	10.39	10.15	10.07	9.85	9.70	9.70	9.60	9.73	9.87	-0.2	(-0.5;0.1)
Distrito Federal	10.46	10.51	10.62	10.73	10.98	11.00	11.01	11.03	11.35	11.27	11.05	10.58	10.47	10.35	10.49	10.13	9.83	9.50	9.33	9.02	8.79	8.71	8.71	8.35	8.11	7.95	7.84	7.64	7.61	7.68	-1.1*	(-1.3;-0.8)
Espírito Santo	8.75	9.00	9.18	9.35	9.45	9.27	9.56	9.33	9.72	9.18	9.51	9.25	8.71	8.31	8.44	8.52	8.64	8.77	8.98	8.99	8.85	8.97	9.05	8.96	9.17	9.11	9.06	9.15	9.19	9.27	0.2	(-0.3;0.7)
Goiás	12.12	12.40	12.41	12.46	12.56	12.77	13.06	13.15	13.05	12.34	11.89	11.96	12.04	12.33	12.39	12.17	12.11	11.61	11.39	11.10	10.94	10.55	10.16	9.80	9.35	9.06	8.76	8.63	8.73	8.81	-1.1*	(-1.4;-0.8)
Maranhão	9.84	9.85	10.06	10.07	9.96	9.84	9.70	9.41	9.25	9.03	9.00	9.04	9.09	9.07	8.80	8.87	8.95	9.07	9.09	9.12	9.33	9.91	10.26	10.57	10.78	10.97	11.05	11.20	11.15	11.17	0.5*	(0.2;0.8)
Mato Grosso	9.80	9.79	9.66	10.22	10.53	10.77	11.07	11.36	11.69	11.95	12.16	12.06	12.32	12.60	12.76	13.10	12.24	11.67	11.52	11.48	11.41	10.96	10.65	10.32	10.12	9.69	9.58	9.42	9.40	9.46	-0.1	(-0.5;0.3)
Mato Grosso do Sul	9.76	9.86	9.79	10.13	10.51	10.55	10.60	10.60	10.77	11.01	10.75	10.61	10.30	10.46	10.69	10.64	10.79	10.78	10.88	10.52	10.62	10.62	10.26	10.20	9.73	9.49	9.45	8.88	8.98	9.04	-0.3	(-0.9;0.3)
Minas Gerais	10.52	10.71	10.78	10.96	11.11	11.19	11.41	11.67	11.84	11.95	12.14	12.30	12.94	12.88	13.01	13.08	12.99	12.83	12.58	12.26	11.92	11.33	10.83	10.38	9.98	9.86	9.64	9.38	9.39	9.45	-0.4*	(-0.7;-0.1)
Pará	8.82	8.79	8.70	8.62	8.81	8.85	8.99	9.01	9.08	9.15	9.48	9.82	9.83	10.00	10.04	10.19	10.10	9.96	9.91	9.85	9.82	9.50	9.29	9.21	8.98	8.78	8.68	8.37	8.37	8.39	-0.2	(-0.4;0.1)
Paraíba	9.86	10.00	10.13	10.27	10.21	10.50	10.52	10.33	10.41	10.23	10.25	10.31	10.52	10.47	10.76	11.18	11.48	11.51	11.73	11.56	11.59	11.62	11.29	11.18	10.86	10.56	10.39	10.33	10.32	10.37	0.2	(-0.2;0.5)
Paraná	9.22	9.04	9.22	9.47	9.70	9.91	10.29	10.54	10.83	10.61	10.48	10.68	10.77	10.75	10.99	10.70	10.67	10.49	10.42	10.14	10.01	9.85	9.65	9.51	9.44	9.39	9.30	9.07	9.15	9.18	0.0	(-0.3;0.3)
Pernambuco	9.43	9.20	9.42	9.67	10.09	10.61	10.85	11.20	11.44	10.98	11.58	11.44	11.42	11.52	11.98	11.82	11.99	11.90	11.80	11.68	11.62	11.61	11.51	11.52	11.50	11.36	11.35	11.30	11.42	11.41	0.6*	(0.3;1.0)
Piauí	9.17	8.93	8.83	8.86	8.77	8.64	8.50	8.39	8.40	8.75	9.15	9.12	9.80	9.95	10.20	10.18	10.11	9.63	9.35	9.37	9.20	9.21	9.33	9.26	9.06	8.95	8.79	8.64	8.61	8.67	-0.2	(-0.5;0.1)
Rio de Janeiro	12.30	12.22	12.43	12.84	13.19	13.24	13.28	13.10	13.15	12.99	12.68	12.72	12.41	12.18	12.21	11.76	11.74	11.85	11.69	11.51	11.36	11.19	10.99	10.95	10.68	10.39	10.60	10.48	10.57	10.63	-0.5*	(-0.6;-0.3)
Rio Grande do Norte	8.07	8.21	8.17	8.14	8.50	8.74	8.94	9.07	9.19	9.43	9.92	10.48	10.68	10.70	10.99	10.85	10.92	10.67	10.11	9.54	9.36	9.33	9.31	9.14	9.09	8.84	8.81	8.77	9.01	9.13	0.5	(-0.1;1.1)
Rio Grande do Sul	12.44	12.45	12.58	12.91	12.94	12.78	12.92	12.77	12.74	12.42	12.39	12.04	11.82	11.67	11.46	11.11	10.79	10.46	10.26	10.11	9.91	9.62	9.34	9.28	8.95	8.58	8.56	8.44	8.65	8.77	-1.2*	(-1.4;-0.9)
Rondônia	9.89	9.37	9.10	9.77	9.31	9.23	9.43	9.99	10.06	10.30	10.61	10.52	10.43	10.53	10.53	10.21	10.27	10.32	10.19	10.09	10.17	9.93	9.60	9.31	9.26	9.08	9.14	9.26	9.35	9.49	-0.1	(-0.6;0.5)
Roraima	11.22	10.53	10.10	10.37	10.34	10.18	10.54	10.95	11.15	11.05	11.10	11.79	11.88	12.14	11.96	11.90	11.91	11.74	11.52	11.47	11.22	11.00	10.79	10.93	10.84	10.74	10.76	10.69	11.02	11.12	-0.0	(-0.4;0.3)
Santa Catarina	9.61	9.51	9.64	9.72	9.94	9.86	9.86	9.57	9.73	9.89	9.87	9.63	9.52	9.61	9.48	9.50	9.44	9.37	9.19	9.21	9.11	8.84	8.65	8.36	8.17	8.14	8.09	8.06	8.16	8.24	-0.6*	(-0.8;-0.3)
São Paulo	11.03	10.88	11.07	11.37	11.79	12.05	12.18	12.20	12.30	12.12	11.95	11.77	11.56	11.56	11.48	11.35	11.41	11.20	11.07	10.90	10.70	10.40	10.05	9.77	9.45	9.17	9.06	8.73	8.60	8.57	-0.9*	(-1.1;-0.6)
Sergipe	11.83	11.95	12.31	12.83	13.30	13.79	13.58	13.59	14.07	14.57	14.66	14.29	14.29	14.42	14.82	14.97	14.67	14.36	13.82	13.20	13.11	12.76	12.72	12.49	11.85	11.14	11.36	11.13	11.18	11.21	-0.2	(-0.5;0.2)
Tocantins	8.89	9.12	9.34	9.48	9.59	9.76	9.95	10.19	10.29	10.23	10.46	10.51	10.88	11.25	11.55	11.54	11.66	11.27	11.24	11.44	11.71	11.80	11.83	11.85	11.88	11.77	11.49	10.96	11.00	10.98	0.7*	(0.2;1.2)
**Brazil**	10.62	10.59	10.73	10.96	11.19	11.33	11.48	11.53	11.62	11.54	11.55	11.50	11.52	11.54	11.59	11.47	11.45	11.31	11.18	11.00	10.85	10.63	10.42	10.23	10.00	9.82	9.74	9.56	9.58	9.62	-0.3*	(-0.5;-0.2)

AAPC = annual average percent change
95% CI= 95% Confidence Interval
*Statistical significance at level of 5%

Supplementary Table 4. Standardized rate of prostate câncer mortality in men of 60-69 years of age, according State and year, 1990-2019.

Brazilian States	Year																															
	**1990**	**1991**	**1992**	**1993**	**1994**	**1995**	**1996**	**1997**	**1998**	**1999**	**2000**	**2001**	**2002**	**2003**	**2004**	**2005**	**2006**	**2007**	**2008**	**2009**	**2010**	**2011**	**2012**	**2013**	**2014**	**2015**	**2016**	**2017**	**2018**	**2019**	**AAPC**	**95%CI**
Acre	50.53	51.97	52.81	52.30	54.41	53.82	55.58	55.40	55.56	55.08	55.69	57.27	56.16	55.25	56.51	56.65	57.55	57.34	56.07	54.83	55.29	54.34	55.20	53.26	51.99	50.81	50.95	51.25	52.04	52.32	0.1	(-0.2;0.4)
Alagoas	54.33	54.20	54.62	54.83	54.55	54.03	53.31	53.18	52.96	52.23	53.92	55.36	56.71	56.95	57.92	57.45	58.00	57.92	57.97	58.32	58.75	58.63	59.16	60.34	57.59	56.81	55.19	54.85	56.13	56.34	0.1	(-0.1;0.4)
Amapá	47.51	49.39	51.41	52.51	53.23	55.12	56.36	56.91	57.00	56.17	56.93	54.91	55.22	55.11	53.56	53.15	52.87	53.05	53.46	53.48	53.19	52.87	54.82	54.63	54.47	53.92	52.78	52.10	52.55	52.52	0.3*	(0.1;0.5)
Amazonas	55.55	55.42	55.13	55.45	54.67	56.26	54.33	54.95	56.51	58.65	59.98	61.97	62.44	62.78	61.78	62.53	60.51	59.76	59.52	57.84	57.54	56.38	55.26	54.70	55.18	55.93	54.86	53.54	54.27	54.93	-0.1	(-0.3;0.2)
Bahia	61.88	63.67	65.84	67.64	69.38	72.40	74.58	75.83	76.75	78.01	77.77	77.48	77.71	77.84	78.46	78.59	79.81	80.82	81.41	80.45	79.96	78.79	79.38	79.58	79.60	80.89	82.15	82.98	82.67	82.82	1.0*	(0.8;1.3)
Ceará	59.82	61.16	61.71	63.47	65.49	68.42	67.81	67.25	64.35	63.39	62.84	62.12	63.23	64.53	65.73	66.10	66.45	65.92	64.61	62.21	59.42	57.95	56.40	55.15	54.16	53.85	53.39	53.11	52.96	53.38	-0.4*	(-0.6;-0.1)
Distrito Federal	67.96	69.45	71.09	71.91	73.92	76.02	77.50	79.69	79.56	79.03	78.86	77.38	75.04	73.29	73.88	72.35	69.90	67.44	66.18	62.93	60.43	58.73	57.22	54.92	54.03	51.86	50.70	48.64	49.56	50.00	-1.0*	(-1.2;-0.8)
Espírito Santo	53.85	55.29	56.40	56.88	57.47	55.00	57.44	57.06	60.22	58.75	59.18	58.99	57.71	56.57	54.88	54.87	54.69	54.89	54.13	52.98	52.45	51.52	50.65	49.66	49.97	49.41	49.24	49.92	49.95	50.66	-0.3*	(-0.5;-0.0)
Goiás	69.87	71.28	70.94	71.78	72.24	72.55	74.95	76.66	77.31	75.89	75.02	75.77	76.04	77.04	75.90	76.41	75.72	74.20	71.28	68.22	66.04	63.65	61.08	58.48	57.97	56.59	55.14	53.94	54.22	54.31	-0.9*	(-1.2;-0.5)
Maranhão	57.37	57.68	58.95	59.30	58.82	58.41	57.32	54.80	53.89	52.87	51.98	51.16	51.78	51.47	50.22	50.76	50.38	50.83	51.47	51.95	53.53	59.02	59.59	60.41	62.68	63.40	64.55	65.92	66.38	66.68	0.5*	(0.2;0.9)
Mato Grosso	57.68	57.49	57.01	58.62	59.77	61.46	63.31	65.55	67.28	74.02	77.83	79.93	80.31	82.08	82.30	82.14	79.79	78.29	76.39	72.31	69.38	66.26	63.35	60.25	59.31	56.23	55.49	53.57	52.91	53.03	-0.3	(-0.7;0.1)
Mato Grosso do Sul	60.63	62.49	62.13	65.01	67.14	68.83	70.26	69.92	69.86	70.56	68.94	67.12	65.82	66.78	68.19	67.97	67.55	65.28	64.10	60.66	60.09	57.30	56.18	54.66	52.86	52.45	52.91	50.37	50.28	50.47	-0.6*	(-1.3;-0.0)
Minas Gerais	57.70	58.10	58.75	59.61	60.14	60.96	62.36	64.07	66.66	67.78	68.98	71.63	73.88	73.65	74.84	74.67	73.90	71.35	69.70	66.59	64.03	60.63	58.18	55.53	53.52	52.35	52.06	51.11	50.50	50.68	-0.5*	(-0.7;-0.2)
Pará	50.88	50.60	50.40	50.05	50.83	50.11	51.57	52.69	53.00	52.94	56.35	56.00	56.24	58.68	59.57	59.34	59.49	60.54	59.89	58.74	58.29	56.10	54.12	53.42	52.15	51.45	52.24	51.42	50.81	50.88	0.0	(-0.2;0.3)
Paraíba	57.55	58.16	57.60	56.40	55.70	57.40	57.69	56.09	56.81	58.45	57.83	56.41	57.49	56.46	60.87	63.52	63.18	63.52	65.47	65.20	65.25	65.28	63.14	61.19	60.13	59.03	59.07	58.63	57.32	57.37	-0.0	(-0.3;0.3)
Paraná	56.64	57.57	59.72	62.27	63.72	64.55	68.11	69.23	70.52	69.55	69.07	69.13	68.90	69.07	68.92	67.35	66.53	65.06	63.80	62.06	60.84	59.95	58.69	57.24	56.06	55.26	54.56	54.02	54.23	54.47	-0.1	(-0.3;0.0)
Pernambuco	59.94	60.07	60.03	62.77	63.80	64.77	64.81	65.73	67.17	66.30	66.76	65.87	67.53	69.25	70.74	71.11	71.43	71.01	69.74	68.44	67.37	66.56	65.93	65.18	64.22	63.89	63.86	62.86	62.83	62.45	0.2	(-0.1;0.5)
Piauí	58.43	57.58	59.43	59.20	57.75	56.62	55.95	57.20	58.59	60.28	62.02	64.00	66.02	64.86	63.82	63.00	61.15	57.22	55.85	56.19	57.00	55.25	56.00	56.11	54.61	54.22	53.21	52.38	52.04	52.28	-0.4*	(-0.8;-0.1)
Rio de Janeiro	70.70	71.56	72.11	74.88	76.59	77.75	79.10	78.48	79.11	79.14	77.87	78.23	77.16	76.50	75.66	73.60	72.64	71.66	68.33	66.04	63.90	62.22	60.59	60.38	59.37	59.26	60.91	60.41	60.39	60.47	-0.5*	(-0.7;-0.3)
Rio Grande do Norte	52.37	54.33	55.43	55.02	56.75	57.88	57.43	55.65	56.60	55.73	59.21	59.58	60.42	62.25	61.99	61.16	62.62	61.81	60.39	57.12	55.81	56.28	55.62	54.97	54.54	53.26	53.29	52.49	52.65	52.55	-0.0	(-0.6;0.5)
Rio Grande do Sul	70.42	71.39	72.44	74.45	76.66	79.20	81.54	81.75	82.41	81.92	82.00	81.20	79.00	78.89	78.09	74.36	72.54	70.05	66.72	64.46	62.45	59.46	58.13	57.74	56.98	55.28	55.14	54.05	55.02	55.46	-0.8*	(-1.0;-0.5)
Rondônia	57.39	55.54	53.68	54.83	51.98	53.73	56.78	58.19	59.43	61.81	64.30	64.51	64.66	64.34	64.09	62.39	62.01	59.89	59.63	58.25	58.15	58.17	59.29	59.13	60.92	62.69	61.76	61.98	61.69	60.94	0.2	(-0.1;0.6)
Roraima	82.61	81.22	78.76	77.86	75.77	72.46	71.03	71.22	70.48	65.25	67.44	67.89	67.55	69.71	69.58	68.71	67.62	63.70	60.16	60.34	59.98	60.23	60.94	60.90	61.58	61.45	62.71	63.80	63.46	63.38	-0.9*	(-1.3;-0.6)
Santa Catarina	61.42	61.94	63.02	63.30	63.78	64.19	64.41	64.27	66.12	66.50	66.92	66.99	65.71	65.75	65.40	63.85	62.05	60.40	58.65	56.99	55.18	52.70	50.65	49.19	48.04	46.94	46.82	46.35	46.98	47.42	-0.9*	(-1.2;-0.6)
São Paulo	65.39	65.26	65.78	67.76	69.53	71.17	73.23	73.66	74.06	74.66	74.97	73.53	72.65	72.70	72.07	70.83	70.03	67.80	66.31	64.55	62.31	60.23	57.99	56.14	54.63	53.56	53.44	51.51	50.62	50.40	-0.9*	(-1.2;-0.7)
Sergipe	69.37	70.04	71.60	72.21	72.23	76.71	76.80	76.53	79.52	79.86	80.94	82.20	83.94	82.44	83.09	83.70	82.98	82.37	79.97	76.77	76.07	73.68	70.57	69.25	66.41	63.04	61.91	60.91	60.78	60.85	-0.4*	(-0.7;-0.1)
Tocantins	56.28	54.25	53.43	53.61	54.16	55.20	57.42	59.83	59.28	60.68	62.32	61.68	62.03	63.59	62.87	65.38	65.74	66.46	65.15	66.38	66.00	65.50	67.16	67.90	68.85	69.29	70.05	67.78	65.82	65.46	0.5*	(0.2;0.9)
**Brazil**	62.45	63.01	63.75	65.31	66.51	67.79	69.21	69.61	70.39	70.63	70.89	70.77	70.72	70.85	70.86	70.04	69.42	68.08	66.61	64.73	63.12	61.40	59.89	58.66	57.59	56.83	56.85	55.92	55.68	55.75	-0.4*	(-0.5;-0.2)

AAPC = annual average percent change
95% CI= 95% Confidence Interval
*Statistical significance at level of 5%

Supplementary Table 5. Standardized rate of prostate câncer mortality in men of 70-79 years of age, according State and year, 1990-2019.

Brazilian States	Year																															
	1990	1991	1992	1993	1994	1995	1996	1997	1998	1999	2000	2001	2002	2003	2004	2005	2006	2007	2008	2009	2010	2011	2012	2013	2014	2015	2016	2017	2018	2019	AAPC	95%CI
Acre	189.51	196.61	199.47	190.56	200.33	203.63	216.29	227.19	223.99	220.44	231.25	235.71	242.59	241.22	254.56	253.11	256.13	245.63	232.93	229.35	227.19	219.70	215.86	207.05	203.33	204.58	206.17	207.29	208.50	209.65	0.4*	(0.1;0.7)
Alagoas	199.93	198.53	197.05	197.37	195.05	192.99	191.54	191.06	191.01	197.05	194.82	194.91	197.90	198.68	202.75	206.15	207.98	207.54	207.89	206.56	206.83	207.34	210.23	210.32	204.38	196.28	189.63	188.86	192.23	192.25	-0.1	(-0.4;0.1)
Amapá	177.67	176.90	175.70	178.93	182.66	187.53	191.81	194.23	196.23	202.41	210.91	212.92	217.43	216.94	210.79	204.56	203.14	199.54	202.03	199.98	201.50	198.62	203.77	207.78	210.51	212.12	210.24	206.71	209.28	208.53	0.5*	(0.2;0.9)
Amazonas	197.77	194.55	201.39	200.91	200.49	203.90	203.58	204.56	214.10	221.99	236.71	240.06	238.77	243.12	241.45	243.01	238.90	234.79	232.38	227.10	219.97	218.20	218.14	220.94	223.99	231.39	229.50	224.15	221.78	221.93	0.4	(-0.0;0.8)
Bahia	214.58	217.49	222.08	228.74	230.72	243.86	249.84	261.21	270.20	276.19	283.49	291.17	292.15	293.66	297.24	298.84	301.37	305.91	306.40	302.08	296.73	295.04	296.13	292.84	290.83	291.17	293.09	290.15	285.65	284.50	1.0*	(0.7;1.3)
Ceará	240.18	241.89	240.70	244.66	254.19	272.08	273.21	273.02	268.27	265.70	267.12	263.40	264.59	266.37	262.80	257.69	254.59	247.21	244.16	233.85	222.36	216.31	211.48	207.37	207.10	207.44	208.58	207.62	206.55	207.52	-0.5*	(-0.8;-0.3)
Distrito Federal	281.24	275.42	275.17	269.33	274.74	281.72	295.44	318.62	317.53	323.87	324.45	328.06	323.02	315.36	298.04	280.20	270.18	263.51	260.05	252.18	247.95	242.34	231.54	222.78	220.14	211.98	208.55	202.83	205.70	206.39	-1.0*	(-1.5;-0.6)
Espírito Santo	184.54	186.82	188.68	190.90	192.47	189.80	193.45	187.96	193.25	191.04	192.85	193.29	194.81	196.57	201.85	204.51	207.25	207.22	205.39	206.21	210.82	210.94	208.41	204.69	208.81	210.07	208.01	208.14	206.96	207.43	0.4*	(0.2;0.6)
Goiás	240.80	243.62	244.60	246.92	248.83	256.25	263.08	273.26	275.66	279.64	276.49	279.98	278.98	275.14	270.18	263.17	261.10	258.57	250.48	245.87	242.62	235.10	225.13	216.59	212.68	209.22	202.94	196.94	198.73	199.01	-0.7*	(-1.0;-0.3)
Maranhão	195.24	197.14	202.16	202.53	199.05	196.13	195.33	193.19	186.98	180.65	177.97	174.15	171.61	169.90	166.13	167.42	167.33	168.66	170.59	172.73	180.49	205.93	212.01	218.57	226.51	226.96	228.09	230.23	232.79	234.18	0.7*	(0.3;1.0)
Mato Grosso	242.55	244.56	236.26	244.84	234.00	243.51	244.98	247.28	255.14	281.55	292.70	293.06	295.31	302.05	302.75	304.45	293.06	280.91	278.18	261.36	252.37	245.63	234.83	227.37	226.41	221.26	218.28	210.61	209.75	210.26	-0.5	(-1.0;0.0)
Mato Grosso do Sul	216.45	214.14	209.62	213.69	217.22	215.77	221.44	225.14	232.32	239.92	239.13	234.35	235.43	243.32	246.68	242.05	241.69	236.77	236.94	233.50	230.58	223.78	216.86	212.97	209.26	207.72	207.11	196.08	194.14	194.08	-0.4*	(-0.7;-0.1)
Minas Gerais	212.38	212.84	218.31	222.92	226.48	227.13	229.42	234.03	242.99	248.28	249.89	257.55	267.91	269.47	271.09	272.39	270.05	262.72	252.97	243.32	233.56	222.22	212.75	204.84	197.99	193.07	188.74	182.64	181.72	181.80	-0.5*	(-0.9;-0.2)
Pará	193.47	190.09	187.54	186.07	183.05	183.86	186.30	188.24	190.87	195.73	204.44	202.33	205.10	210.65	215.16	214.56	216.89	219.69	217.89	217.80	219.63	209.62	203.69	202.72	200.12	194.72	194.24	191.79	188.94	188.16	-0.1	(-0.3;0.0)
Paraíba	214.77	218.31	217.08	214.88	220.11	231.13	238.67	241.20	235.86	238.39	242.39	234.20	232.57	229.21	240.79	243.39	245.29	248.55	254.57	243.92	239.17	232.16	220.87	210.23	206.23	205.69	201.46	197.97	194.22	194.96	-0.4	(-0.8;0.1)
Paraná	196.60	196.93	201.19	210.78	213.73	216.80	229.36	235.43	248.01	251.97	254.96	257.65	260.51	264.04	263.55	255.30	256.17	251.81	247.12	244.25	241.37	236.87	232.50	228.38	223.49	220.78	217.45	213.66	213.26	213.37	0.3	(-0.0;0.6)
Pernambuco	200.10	197.31	198.23	207.89	208.42	210.47	218.11	227.70	238.48	244.58	252.58	258.36	266.68	273.32	273.45	269.23	264.79	262.31	259.47	257.91	253.18	249.77	246.08	244.14	244.94	245.44	246.31	246.13	243.77	241.07	0.7*	(0.6;0.9)
Piauí	216.55	212.95	213.51	210.93	206.57	203.67	202.84	213.73	220.56	226.90	237.76	254.07	256.61	247.25	236.05	230.68	224.82	211.94	204.94	200.08	202.85	194.16	196.27	189.07	181.94	178.14	176.29	174.73	174.06	174.78	-0.8*	(-1.2;-0.3)
Rio de Janeiro	250.56	253.80	256.40	262.39	265.60	263.34	262.56	262.22	266.88	267.25	265.44	268.94	270.20	269.90	269.27	264.85	264.63	264.53	259.35	252.30	245.87	237.40	230.70	228.36	222.39	219.01	221.66	218.19	218.10	217.28	-0.5*	(-0.7;-0.3)
Rio Grande do Norte	189.85	193.59	193.23	195.83	201.52	198.98	199.92	204.35	213.05	214.57	217.07	224.24	234.43	244.42	244.25	248.18	253.83	246.97	231.92	218.06	211.26	209.42	206.46	203.71	203.35	202.88	206.03	204.08	202.18	202.05	0.2	(-0.1;0.5)
Rio Grande do Sul	265.72	267.78	272.39	281.03	282.47	284.16	288.65	288.85	293.13	292.09	290.88	287.67	283.95	283.01	280.36	275.28	272.91	269.22	261.36	255.64	249.53	239.77	234.10	229.27	223.09	214.15	213.37	208.01	210.03	210.49	-0.8*	(-1.0;-0.6)
Rondônia	241.24	221.66	214.98	213.45	212.84	219.02	219.16	224.03	229.60	230.63	233.08	228.04	232.17	240.36	238.20	238.61	236.97	230.57	233.33	231.39	227.86	222.72	227.42	224.29	227.39	233.62	231.16	229.52	227.90	226.78	-0.1	(-0.5;0.2)
Roraima	306.87	304.75	300.14	296.90	296.71	294.31	291.36	298.06	297.60	287.16	295.87	289.20	292.59	296.76	281.18	269.74	264.44	256.42	243.18	246.62	239.36	234.02	235.93	229.47	218.95	222.58	222.90	228.63	226.22	225.94	-1.0*	(-1.2;-0.7)
Santa Catarina	216.72	218.78	222.66	226.22	231.82	233.80	235.07	239.33	246.47	252.55	252.48	254.67	252.26	250.09	250.01	244.30	240.42	238.17	233.36	229.62	225.81	215.04	205.56	200.09	197.70	194.86	192.44	187.84	189.40	190.22	-0.4*	(-0.7;-0.2)
São Paulo	239.00	237.14	237.96	244.00	247.56	251.30	256.70	260.65	264.47	267.24	266.40	264.30	261.34	261.51	258.54	251.41	248.29	241.04	235.58	230.80	222.89	214.90	206.91	201.29	196.17	192.45	189.65	182.37	180.48	179.37	-1.0*	(-1.2;-0.8)
Sergipe	245.49	243.12	248.18	257.13	263.55	270.10	266.55	267.07	277.49	281.82	292.89	293.08	310.24	316.14	307.25	302.64	303.01	309.18	301.38	290.91	283.45	269.31	264.41	263.12	252.48	242.76	239.53	239.20	237.52	236.88	-0.1	(-0.4;0.3)
Tocantins	193.04	183.61	182.06	182.01	179.22	180.94	186.31	188.46	188.71	192.36	185.89	193.10	196.24	203.70	203.46	209.93	216.91	216.55	214.11	219.77	228.64	228.50	235.25	244.65	259.78	255.70	250.56	241.70	237.56	234.92	0.7*	(0.2;1.3)
**Brazil**	224.71	225.13	227.33	232.44	235.16	238.82	242.84	246.85	251.90	254.94	256.67	258.28	259.68	260.71	259.94	256.51	254.98	251.22	246.50	241.05	235.55	229.43	223.84	219.44	215.81	212.69	211.04	206.51	205.44	204.98	-0.3*	(-0.5;-0.2)

AAPC = annual average percent change
95% CI= 95% Confidence Interval
*Statistical significance at level of 5%

Supplementary Table 6. Standardized rate of prostate câncer mortality in men ≥80 years of age, according State and year, 1990-2019.

Brazilian States	Year																														AAPC	95%CI
	1990	1991	1992	1993	1994	1995	1996	1997	1998	1999	2000	2001	2002	2003	2004	2005	2006	2007	2008	2009	2010	2011	2012	2013	2014	2015	2016	2017	2018	2019		
Acre	798.72	789.03	784.23	762.18	698.59	632.65	650.47	658.36	625.00	602.95	615.76	616.55	606.36	604.39	637.56	646.02	681.26	688.12	702.06	705.62	738.39	770.76	791.24	790.84	782.14	762.33	738.17	728.11	735.61	734.27	-0.3	(-1.0;0.4)
Alagoas	473.03	468.86	466.64	460.14	452.55	445.15	435.39	428.42	429.34	457.95	456.01	447.18	457.38	475.68	489.77	497.03	499.91	517.05	513.26	518.02	509.58	498.89	514.56	531.69	547.67	551.74	539.82	535.61	530.22	527.11	0.3	(-0.3;1.0)
Amapá	632.28	677.18	668.35	668.57	690.97	695.26	679.91	640.85	633.90	623.52	608.40	611.63	641.02	619.79	607.52	592.43	602.69	611.08	633.00	618.86	601.75	602.71	619.10	640.99	650.84	656.37	667.66	645.66	634.61	631.08	-0.1	(-0.9;0.7)
Amazonas	557.75	536.81	537.44	539.52	553.67	534.97	506.47	503.78	524.39	571.29	586.72	583.10	590.34	588.17	593.13	603.69	591.49	589.86	585.95	565.61	567.43	567.03	569.37	572.54	578.45	587.92	603.54	602.25	589.76	599.49	0.3	(-0.2;0.9)
Bahia	519.81	526.80	537.19	552.69	552.21	552.35	559.19	572.26	588.87	611.10	635.63	657.15	686.91	701.34	734.42	778.03	820.76	844.29	860.86	868.75	855.36	826.65	827.77	817.76	801.74	793.30	788.33	781.29	764.34	752.71	1.3*	(1.2;1.4)
Ceará	614.75	624.80	632.85	643.36	660.84	702.38	720.55	728.31	699.21	670.15	670.99	641.54	637.40	656.41	670.67	677.67	690.21	695.09	690.80	692.33	691.43	671.46	671.72	672.72	682.66	694.01	704.48	698.21	668.79	665.78	0.3	(-0.3;0.9)
Distrito Federal	1173.27	1137.89	1044.68	967.12	934.79	872.49	856.92	849.50	803.23	744.90	704.47	756.49	795.68	792.58	801.72	820.35	845.65	878.54	926.87	971.39	993.94	966.34	923.36	886.47	865.85	826.57	791.61	763.99	788.64	788.52	-1.3*	(-1.8;-0.7)
Espírito Santo	485.67	480.01	473.33	475.97	471.03	455.62	465.66	458.75	481.38	477.39	485.18	489.80	491.52	515.41	548.18	534.20	543.11	551.15	563.49	578.42	593.67	607.84	624.24	627.55	645.52	662.44	676.09	667.29	650.64	648.74	1.0*	(0.7;1.3)
Goiás	594.57	578.99	586.63	601.22	609.00	637.04	670.68	687.85	708.40	723.81	729.98	728.58	701.45	691.33	711.14	714.09	710.16	689.52	661.60	656.07	657.78	633.62	620.64	604.90	597.56	590.74	580.23	562.38	570.70	574.04	-0.1	(-0.5;0.3)
Maranhão	561.63	551.22	551.78	536.93	512.15	512.44	569.36	640.82	628.63	613.26	583.80	535.39	485.42	532.27	441.75	404.23	408.33	452.21	503.35	542.81	632.17	924.58	901.30	886.90	872.69	851.13	837.36	827.42	816.85	811.37	1.5*	(0.1;2.9)
Mato Grosso	600.44	612.84	604.67	587.91	568.69	579.71	589.91	610.77	628.23	647.34	667.78	675.42	670.69	686.66	696.21	697.91	685.32	677.19	679.73	669.90	671.31	640.22	624.95	620.30	621.33	616.68	609.14	582.61	574.83	575.62	-0.2	(-0.5;0.0)
Mato Grosso do Sul	539.15	538.96	554.07	569.80	594.76	605.58	629.62	637.47	659.14	676.17	677.60	664.70	671.88	699.22	696.35	683.00	682.03	676.40	680.24	661.86	656.09	634.91	637.17	631.48	620.05	615.38	612.25	596.82	591.36	595.28	0.4*	(0.2;0.6)
Minas Gerais	537.46	527.10	523.92	523.83	522.43	519.31	527.90	539.67	564.02	578.59	627.15	677.32	691.87	655.93	658.79	676.70	671.02	661.25	648.28	638.12	625.60	591.23	576.99	556.64	535.62	531.63	523.37	506.95	507.32	511.35	-0.2	(-0.6;0.3)
Pará	470.04	465.31	465.53	472.09	474.79	465.13	471.58	472.84	472.74	495.28	521.47	518.02	541.41	573.23	571.19	529.66	523.02	518.82	529.30	549.20	564.23	535.11	521.83	528.58	530.11	527.51	528.52	521.53	515.02	512.50	0.3	(-0.1;0.7)
Paraíba	548.65	549.95	546.32	538.70	586.96	624.83	631.59	651.50	663.41	675.76	678.83	635.04	602.24	616.56	656.14	678.61	681.88	675.75	700.08	665.28	650.70	625.86	586.36	570.44	574.45	578.94	577.83	569.13	562.08	565.24	0.1	(-0.8;1.0)
Paraná	530.30	525.63	536.43	551.19	556.45	568.21	588.43	607.46	623.95	637.03	649.46	660.96	662.75	670.40	683.80	664.38	657.27	652.23	655.00	664.96	669.72	660.40	663.55	662.44	652.03	650.15	646.50	639.25	632.36	633.58	0.6*	(0.3;1.0)
Pernambuco	496.23	492.53	485.22	505.95	504.85	510.82	529.29	548.50	567.54	580.08	582.36	584.34	615.23	644.88	668.58	669.49	680.33	688.79	703.64	704.53	708.58	718.03	705.76	704.85	707.12	722.06	726.40	726.23	691.91	683.47	1.1*	(0.7;1.5)
Piauí	552.93	529.32	537.50	532.06	511.31	494.37	508.14	586.39	620.97	656.54	725.22	728.78	740.81	722.38	664.86	650.82	632.75	578.39	549.31	568.16	613.20	577.01	568.36	538.57	509.39	498.78	503.24	515.95	510.45	516.21	-0.3	(-1.2;0.7)
Rio de Janeiro	587.64	593.82	603.59	627.56	647.51	652.76	663.55	666.96	684.79	690.37	691.53	708.48	711.62	712.78	711.76	697.96	688.57	690.25	680.02	665.36	651.53	624.38	616.50	614.19	601.46	600.54	618.27	612.22	607.64	610.12	0.2	(-0.1;0.4)
Rio Grande do Norte	503.30	517.14	525.41	540.26	546.90	535.54	536.26	528.58	535.03	538.89	538.66	573.23	589.82	609.77	625.44	660.13	699.53	721.90	694.72	668.88	644.37	609.58	600.28	597.31	607.93	619.10	620.56	620.23	615.26	617.64	0.7*	(0.4;1.1)
Rio Grande do Sul	677.42	680.92	696.07	715.48	716.52	717.71	724.60	728.13	751.94	752.55	735.94	733.35	731.59	727.79	736.67	719.35	709.98	704.13	687.91	680.99	680.46	655.49	647.67	639.95	632.55	624.69	632.17	622.09	621.61	624.47	-0.3*	(-0.5;-0.1)
Rondônia	778.89	529.71	488.94	604.44	871.38	906.87	765.65	709.26	659.45	613.07	618.72	603.17	544.66	566.75	569.00	572.54	569.32	554.14	555.75	553.14	566.32	582.50	571.49	585.31	625.06	625.24	621.87	627.71	641.94	649.77	-0.4	(-1.6;0.8)
Roraima	1031.91	1072.64	1055.75	989.41	1003.22	1017.43	993.85	950.96	950.23	856.63	846.68	848.04	839.27	827.41	830.61	823.89	831.78	861.78	835.51	813.93	834.98	838.04	835.05	808.55	775.67	752.39	741.41	749.02	768.46	775.39	-1.0*	(-1.7;-0.3)
Santa Catarina	607.15	624.07	627.06	628.28	640.45	647.65	664.22	677.60	703.27	694.13	705.50	720.17	718.72	719.19	727.21	707.37	694.05	676.28	657.73	653.72	644.86	618.78	592.56	581.27	576.92	575.21	573.58	565.29	572.77	579.71	-0.2	(-0.6;0.2)
São Paulo	601.08	597.63	606.65	626.09	644.27	659.64	684.17	699.86	717.38	723.97	717.44	718.35	715.28	709.77	698.18	667.97	647.78	629.83	615.19	603.27	585.90	563.46	556.27	548.68	544.92	542.85	545.28	535.34	535.60	539.55	-0.4*	(-0.6;-0.2)
Sergipe	854.10	839.50	811.06	763.55	716.03	671.33	621.63	623.93	655.18	671.20	674.23	684.36	724.14	766.77	796.45	757.29	761.33	801.01	790.19	748.57	724.16	702.39	724.71	713.62	688.58	642.61	628.83	620.51	631.71	638.43	-1.3*	(-1.7;-0.9)
Tocantins	676.53	631.99	614.40	587.35	546.64	537.46	531.47	525.11	515.61	510.65	492.03	492.55	497.95	516.73	556.41	623.18	646.14	576.82	557.76	598.95	689.24	720.27	750.17	867.42	977.45	962.69	948.51	927.82	888.33	874.43	0.9*	(0.0;1.9)
Brazil	568.71	567.37	571.92	582.47	589.95	597.10	610.58	623.98	637.43	645.06	653.73	659.48	663.70	668.25	672.70	668.79	668.17	665.61	661.67	657.29	654.08	643.82	634.80	627.00	620.12	616.88	616.79	607.08	600.63	601.06	0.2*	(0.0;0.4)

AAPC = annual average percent change
95% CI= 95% Confidence Interval
*Statistical significance at level of 5%
